# Macitentan attenuates cardiovascular remodelling in infant rats with chronic lung disease

**DOI:** 10.1186/s12967-022-03281-2

**Published:** 2022-02-05

**Authors:** Philipp Baumann, Francesco Greco, Susanne Wiegert, Sven Wellmann, Giovanni Pellegrini, Vincenzo Cannizzaro

**Affiliations:** 1 Department of Intensive Care Medicine and Neonatology, University Children’s Hospital Zurich, Zurich, Switzerland; 2 Children’s Research Centre, University Children’s Hospital Zurich, Zurich, Switzerland; 3 Zurich Centre for Integrative Human Physiology, University of Zurich, Zurich, Switzerland; 4 Department of Neonatology, University of Basel Children’s Hospital (UKBB), Basel, Switzerland; 5 Department of Neonatology, University Children’s Hospital Regensburg (KUNO), University of Regensburg, Regensburg, Germany; 6 Laboratory for Animal Model Pathology (LAMP), Institute of Veterinary Pathology, University of Zurich, Zurich, Switzerland; 7 Department of Neonatology, University Hospital Zurich, University of Zurich, Frauenklinikstrasse 10, 8091 Zurich, Switzerland

**Keywords:** Chronic lung disease, Endothelin receptor blockers, Preterm, Rats, Infants, Bronchopulmonary dysplasia

## Abstract

**Background:**

Cardiovascular impairment contributes to increased mortality in preterm infants with chronic lung disease. Macitentan, an endothelin-1 receptor antagonist, has the potential to attenuate pulmonary and cardiovascular remodelling.

**Methods:**

In a prospective randomized placebo-controlled intervention trial, Sprague–Dawley rats were exposed to 0.21 or 1.0 fraction of inspired oxygen (FiO_2_) for 19 postnatal days. Rats were treated via gavage with placebo or macitentan from days of life 5 to 19. Alveoli, pulmonary vessels, *α*-smooth muscle actin content in pulmonary arterioles, size of cardiomyocytes, right to left ventricular wall diameter ratio, and endothelin-1 plasma concentrations were assessed.

**Results:**

FiO_2_ 1.0 induced typical features of chronic lung disease with significant alveolar enlargement (p = 0.012), alveolar (p = 0.048) and pulmonary vessel rarefaction (p = 0.024), higher *α*-smooth muscle actin content in pulmonary arterioles (p = 0.009), higher right to left ventricular wall diameter ratio (p = 0.02), and larger cardiomyocyte cross-sectional area (p < 0.001). Macitentan treatment significantly increased pulmonary vessel count (p = 0.004) and decreased right to left ventricular wall diameter ratios (p = 0.002). Endothelin-1 plasma concentrations were higher compared to placebo (p = 0.015). Alveolar number and size, *α*-smooth muscle actin, and the cardiomyocyte cross-sectional area remained unchanged (all p > 0.05).

**Conclusion:**

The endothelin-1 receptor antagonist macitentan attenuated cardiovascular remodelling in an infant rat model for preterm chronic lung disease. This study underscores the potential of macitentan to reduce cardiovascular morbidity in preterm infants with chronic lung disease.

## Background

Preterm birth interrupts physiological lung development in the saccular phase with immature alveolarisation and compromised vasculogenesis leading to impaired gas exchange [1]. Thus, extremely preterm babies suffer respiratory distress with the necessity of oxygen supplementation and mechanical respiratory support [2]. Over the last decades, prenatal prevention and postnatal treatment of respiratory distress have seen many data-driven improvements including antenatal steroids, surfactant application, non-invasive continuous positive airway pressure, and caffeine citrate treatment [2–5]. However, the incidence of long term sequelae affecting alveolar and pulmonary vascular structures is rising. Thus, the chronic lung disease bronchopulmonary dysplasia (BPD) develops in 32 to 59% of children born before 29 weeks of gestational age [6, 7]. BPD is responsible for longer postnatal hospitalisations, higher frequency of readmissions, and long-term lung function impairment [8, 9]. Additionally, cardiovascular remodelling with decreased pulmonary capillary density and increased arterial wall thickness contributes to poor outcomes [10]. Between 23 and 39 percent of patients with a combination of BPD and pulmonary hypertension (BPD-PH) die already within the first year of life [11–13]. Moreover, BPD-PH is associated with prolonged time on the respirator, longer oxygen dependency, higher tracheostomy rates, and higher frequency of readmissions to the intensive care setting compared to BPD alone [14]. Recently, it has been shown that echocardiographic right ventricular performance markers significantly worsen already on the 7th day of life (DOL) in very preterm infants who will later be diagnosed with BPD [15].

Endothelin-1 (ET-1) receptor antagonists offer a promising field of research in preterm chronic lung disease [16, 17]. ET-1 is thought to be related to major features of preterm chronic lung disease such as lung fibrosis, impaired alveolarization, and diminished angiogenesis [18, 19]. Further, ET-1 increases pulmonary vascular resistance and right ventricular afterload with consecutive right ventricular hypertrophy (RVH) [20, 21]. Therefore, an intervention at the ET-1 receptor level might have a positive effect on pulmonary vascular resistance and right ventricular remodelling. In 2021, ET-1 receptor antagonists found their way into BPD-PH treatment recommendations for children [22]. However, the influence of pharmacological ET-1 receptor blockade on cardiovascular remodelling in the context of BPD has neither been examined in human patients nor in animal models. The aim of this study was to prospectively evaluate the potential of the dual ET-1 receptor antagonist (ET_A_ and ET_B_ receptor) macitentan to mitigate alveolar and cardiovascular remodelling using the established in-vivo hyperoxia BPD infant rat model [23–25]. We hypothesised that oral administration of macitentan attenuates hyperoxia-induced alveolar rarefaction and enlargement, vascular fibrosis, vascular rarefaction, and right ventricular hypertrophy.

## Methods

### Animals

The research protocol, approved by the Cantonal Veterinary Office of Zurich (licence number 95/2014), was conducted according to the Ethical Principles and Guidelines for Experiments on Animals of the Swiss Academy of Medical Sciences and the Swiss Academy of Sciences. Pregnant Sprague Dawley (SD) dams were delivered by Charles Rivers Laboratories International, Inc. (Sulzfeld, Germany) on day 14 of pregnancy.

Newborn rats were randomized to the following study groups: (1) FiO_2_0.21–placebo (0.9% saline solution), (2) FiO_2_0.21-macitentan (Actelion, Allschwil, Switzerland; 30 mg/kg), (3) FiO_2_1.0–placebo, and (4) FiO_2_1.0-macitentan (FiO_2_: fraction of inspired oxygen). Inhaled oxygen concentrations (FiO_2_ 0.21 and FiO_2_ 1.0) were chosen based on our previous dose-finding experiments [24]. Placebo and macitentan were administered daily via oral gavage from DOL 5–19. Macitentan was reconstituted with a gel containing 7.5% of methyl cellulose (Sigma-Aldrich, St. Louis, USA) according to manufacturer’s advice for oral gavage.

From DOL 1 to 19, rats in the FiO_2_1.0 groups were permanently exposed within their individual ventilated cages (T1500 IVC) to FiO_2_ of 1.0, while control animals were held at FiO_2_ 0.21. Oxygen (O_2_) concentrations in cages were monitored with a computer-controlled O_2_-system based on the software IOX (EMKA Technologies, Paris, France). Carbon dioxide (CO_2_) concentrations were kept below 0.4% and controlled via adjusting gas in- and outflow (3–5 L per Minute). O_2_ and CO_2_ concentrations were monitored three times per day using the O_2_ and CO_2_ Datex-Ohmeda sensor (Anandic Medical System, Switzerland). The pups were assessed for health and discomfort checks (i.e. hunched posture, piloerection, eye discharge, and reduced social interaction) three times daily and all findings were recorded on standardised score sheets. On DOL 5 each pup was tattooed by finger skin pricking using the universal rodent numbering system (Aramis Laboratory Animal Microtattoo System, Ketchum Manufacturing, Brockville, Canada).

On DOL 19 infant rats underwent brief inhalational anaesthesia with isoflurane followed by an intraperitoneal injection of 75 μg/g body weight (BW) of ketamine and 10 μg/g BW of xylazine. Once sufficient depth of anaesthesia was confirmed by absence of pedal withdrawal reflex, sternotomy was performed for complete blood evacuation via direct cardiac puncture, the cause of death for infant rats. Dams were culled via CO_2_. Blood was collected in EDTA plastic tubes and kept on ice before centrifugation at 3000 rpm for 10 min. Plasma was frozen at − 80 °C for further analysis of ET-1 via fluorescence immunoassays. Details on tissue processing on DOL 19 were published in detail previously [24].

### Histomorphometry for alveolar remodelling

For each animal, 10 representative pictures of lung regions without large bronchi were taken at × 40 magnification from haematoxylin–eosin (H&E) stained lung sections. Alveolar diameters, equal to the mean interalveolar distance, were calculated via the mean linear intercept (chord) length (Lm) [26]. A grid with 11 parallel lines was fitted to each picture, and the length of each chord was defined by the intercept with the alveolar walls. Mean Lm was calculated by dividing the total length of the line drawn across the lung section by the number of intercepts encountered.

For alveolar counts, in each animal 15 regions of interest (ROIs) with a size of 0.298 mm^2^ were randomly selected from the lung parenchyma to be analysed at × 40 magnification with Visiopharm™ software. The number of alveoli per field in the H&E-stained sections was counted. A threshold classification allowed to distinguish between alveolar lumina and alveolar wall, and to calculate the alveolar count in each ROI.

### Pulmonary artery medial wall thickness and count of pulmonary vessels

We assessed pulmonary arterial medial wall hypertrophy at × 40 magnification in lung sections with anti-*α* smooth muscle actin (SMA) immunostaining. Fifteen ROIs (2.605 mm^2^), containing vessels with a diameter of < 100 µm, were randomly selected from lungs, avoiding areas with terminal bronchioles. A threshold classification allowed to distinguish between *α*-SMA–positive and negative tissue. The results were expressed as *α*-SMA–positive area per cross sectional vessel. Von Willebrand Factor immunostaining allowed the count of pulmonary vessels within the outlined ROIs. A threshold classification allowed to select pulmonary vessels with a diameter between 30 and 100 µm.

### Right ventricular hypertrophy

In H&E-stained heart sections the dimension of the right and left ventricular free wall was measured with the NDP view software (Hamamatsu Photonics), and the right ventricular/left ventricular (RV/LV) ratio was calculated. In addition, in heart sections stained for anti-WGA (wheat germ agglutinin) the cross-sectional area of cardiomyocytes was assessed at × 40 magnification to obtain a second marker of ventricular hypertrophy. A threshold classification allowed the recognition of WGA-stained membrane and empty sarcoplasm in at least 40 representative right ventricular cardiomyocytes with a central 4′,6-diamidino-2-phenylindole (DAPI)-stained nucleus.

### Statistical analysis

For group comparisons two-way ANOVA and t-test were used. Results are expressed as means ± standard deviation for body weight, and as means ± standard error of means for all others results. Statistically significant data are expressed as vertical box plots with median, 10th, 25th, 75th, and 90th percentiles, statistical significance was set at p < 0.05.

## Results

### Well-being and postnatal growth

**Fig. 1:**
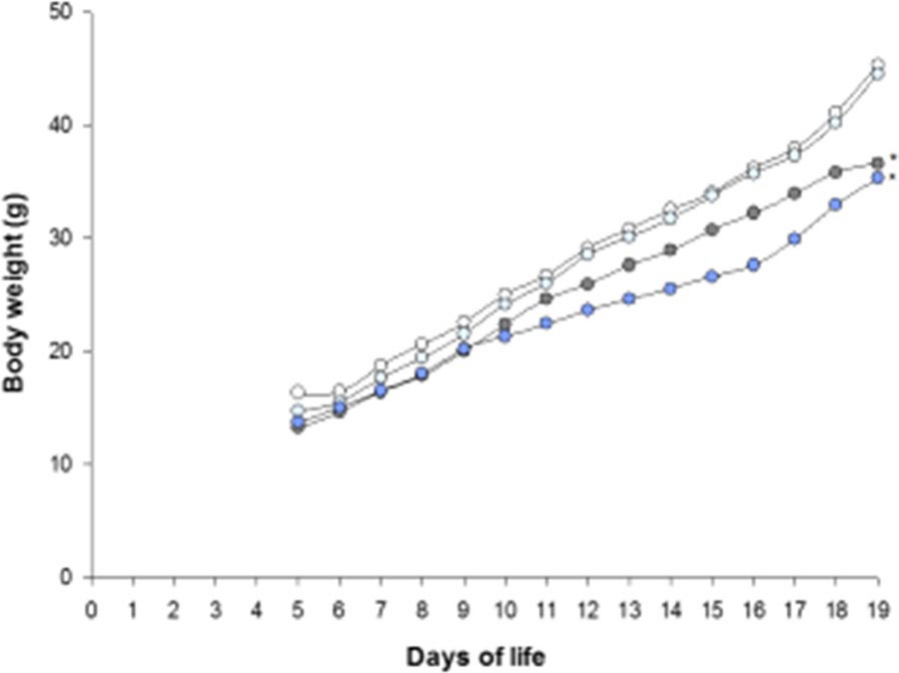
Body weight gain from DOL 5 to 19. White circles: FiO_2_0.21-placebo n = 9, light blue circles: FiO_2_0.21-macitentan n = 10, grey circles: FiO_2_1.0-placebo n = 6, and dark blue circles FiO_2_1.0-macitentan n = 8. *displays significant difference on DOL 19 compared to the corresponding group in FiO_2_0.21, p < 0.05

All animals survived the study protocol showing no signs of stress. FiO_2_ 1.0 led to significantly lower body weight on DOL 19 in both, placebo (p = 0.005) and macitentan (p < 0.001) groups. In contrast, body weight on DOL 19 was not influenced by macitentan neither in FiO_2_ 0.21 (p = 0.724) nor in FiO_2_ 1.0 (p = 0.544) (Fig. 1).

## Histology

### Lung morphometric analysis

**Fig. 2:**
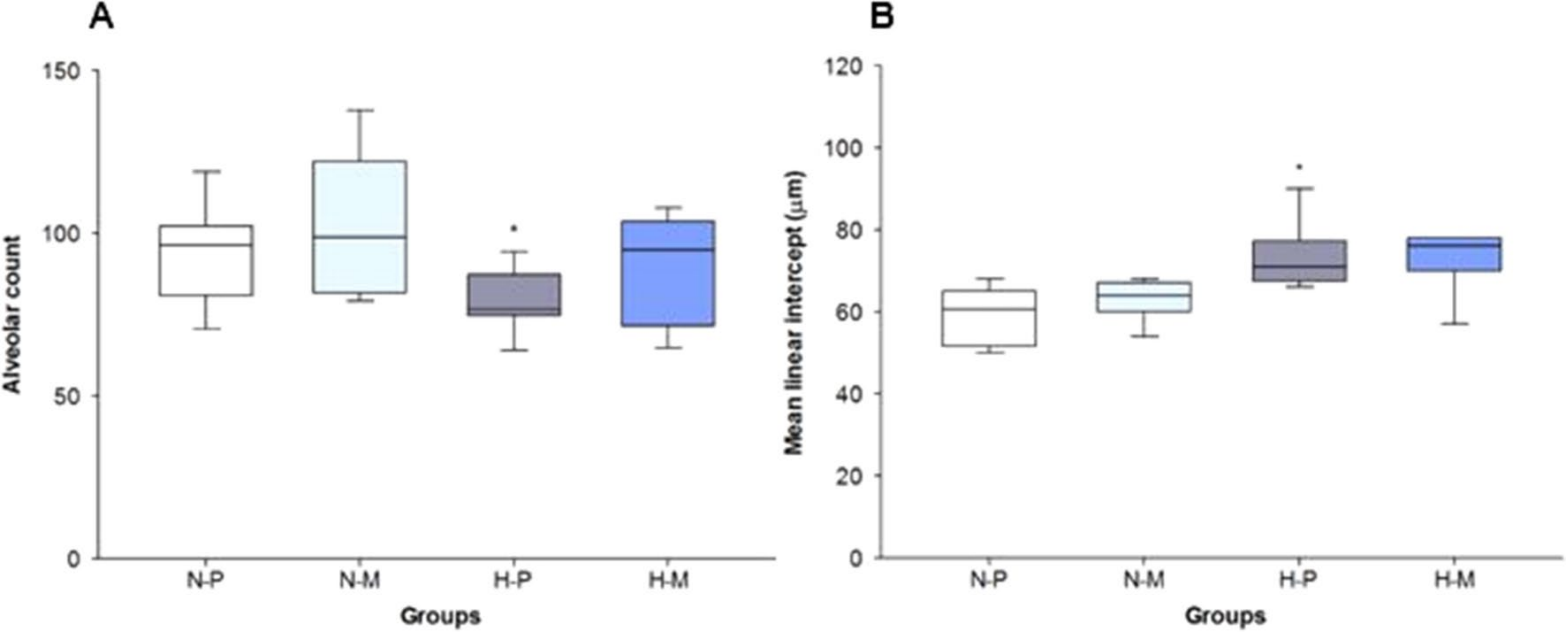
Alveolar count (**A**) and mean linear intercept (**B**) in N-P: normoxia (FiO_2_0.21)-placebo (**A** n = 7, **B** n = 6); N-M: normoxia (FiO_2_0.21)-macitentan (**A** n = 8, **B** n = 9); H-P: hyperoxia (FiO_2_1.0)-placebo (**A** n = 7, **B** = 6); and H-M: hyperoxia (FiO_2_1.0)-macitentan (**A** n = 7, **B** n = 7). Data are expressed as vertical box plots with median, 10th, 25th, 75th, and 90th percentiles. * displays significant difference to FiO_2_0.21-placebo group, p < 0.05

In comparison with FiO_2_ 0.21, FiO_2_ 1.0 led to a significantly lower alveolar count (p = 0.048) and higher mean alveolar intercept in placebo animals (p = 0.012). However, administration of macitentan to infant rats exposed to FiO_2_ 1.0 did not determine significant changes in alveolar count (p = 0.165) or diameter (p = 0.923) in comparison to FiO_2_1.0-placebo (Fig. 2).

### Count of pulmonary vessels

**Fig. 3:**
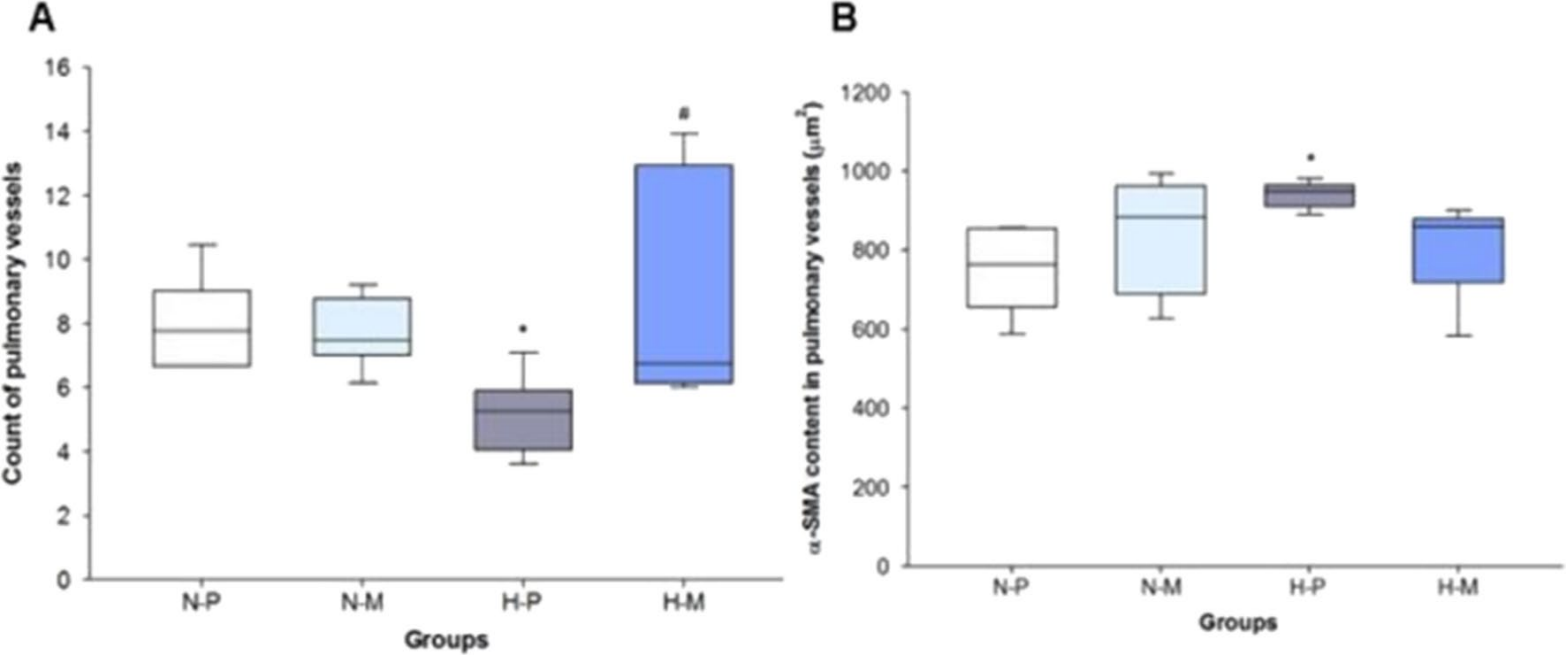
Count of pulmonary vessels (**A**) and *α*-SMA content in the medial wall of the pulmonary arterioles (**B**) and in N-P: normoxia (FiO_2_ 0.21)-placebo (**A** n = 6, **B** n = 6); N-M: normoxia (FiO_2_ 0.21)-macitentan (**A** n = 9, **B** n = 8); H-P: hyperoxia (FiO_2_ 1.0)-placebo (**A** n = 5, **B** n = 6); and H-M: hyperoxia (FiO_2_ 1.0)-macitentan (**A** n = 5, **B** n = 7). Data are expressed as vertical box plots with median, 10th, 25th, 75th, and 90th percentiles. * and ^#^ indicate significant difference to FiO_2_ 0.21-placebo and FiO_2_ 1.0-placebo, respectively (p < 0.05)

FiO_2_ 1.0 led to significantly lower counts of pulmonary vessels compared to FiO_2_ 0.21 in placebo treated animals (p = 0.024). Administration of macitentan in FiO_2_ 1.0 resulted in a significantly higher number of pulmonary vessels when compared with FiO_2_1.0-placebo (p = 0.004; Fig. 3).

### *α*-SMA content in the medial wall of the pulmonary arterioles

**Fig. 4:**
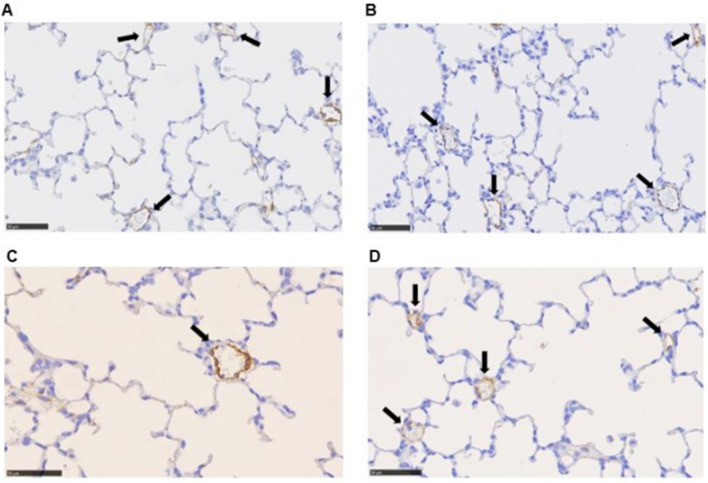
Representative microscopic photographs of anti-*α*-SMA immunostained lung sections of (**A**) FiO_2_ 0.21-placebo, (**B**) FiO_2_ 0.21-macitentan, (**C**) FiO_2_ 1.0-placebo, and (**D**) FiO_2_ 1.0-macitentan groups, respectively. Black arrows indicate cross sections of pulmonary vessels. Scale bar = 50 µm

The FiO_2_1.0-placebo group showed significantly higher *α*-SMA levels in the medial wall of pulmonary arterioles compared to FiO_2_ 0.21 (p = 0.009). Treatment with macitentan had no effect on *α*-SMA content in pulmonary arterioles in FiO_2_ 0.21 (p = 0.365) and FiO_2_ 1.0 (p = 0.064) (Fig. 3). Representative microscopic photographs of anti-*α*-SMA immunostained lung sections: Fig. 4.

### Right to left ventricle diameter ratio

**Fig. 5:**
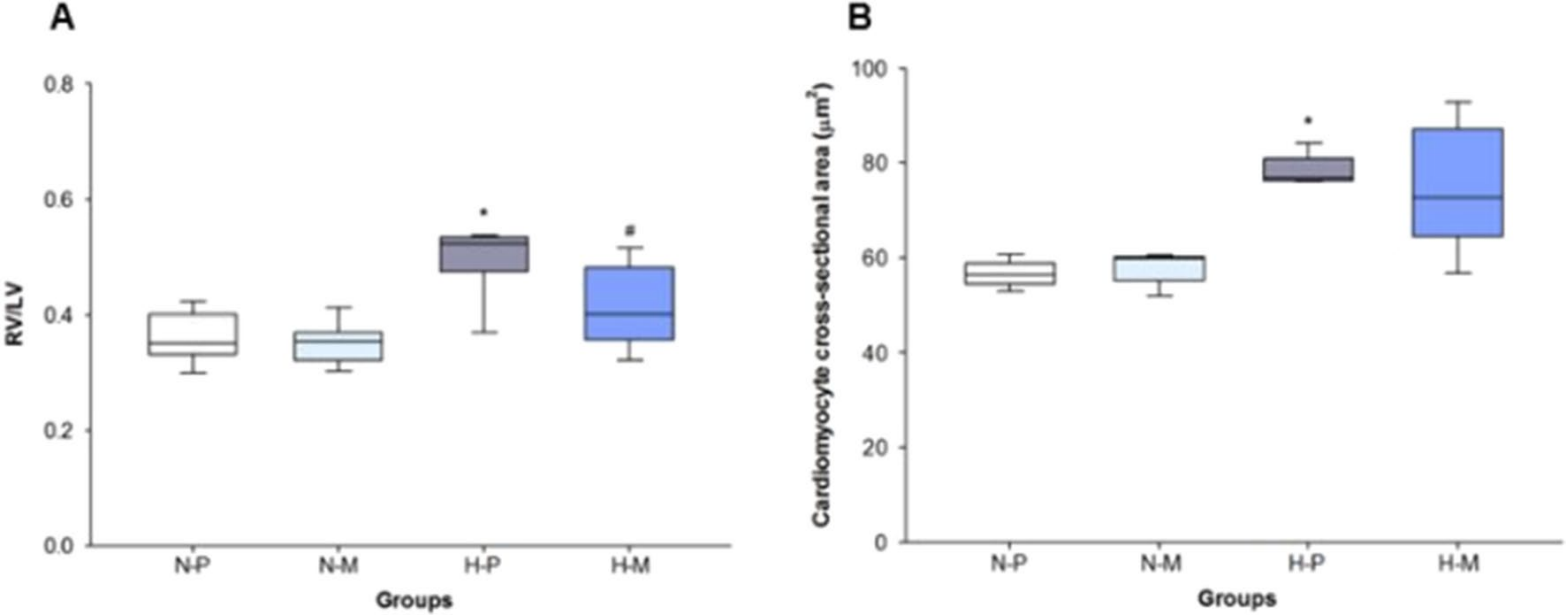
Right-to-left ventricle diameter ratio (A) and cardiomyocyte cross-sectional area (**B**) in N-P: normoxia (FiO_2_ 0.21)-placebo (**A** n = 7, **B** n = 5); N-M: normoxia (FiO_2_ 0.21)-macitentan (**A** n = 9, **B** n = 5); H-P: hyperoxia (FiO_2_ 1.0)-placebo (**A** n = 7, **B** n = 5); and H-M: hyperoxia (FiO_2_ 1.0)-macitentan (**A** n = 7, **B** n = 5). Data are expressed as vertical box plots with median, 10th, 25th, 75th, and 90th percentiles. * and ^#^ indicate significant difference to FiO_2_ 0.21-placebo and FiO_2_ 1.0-placebo, respectively, p < 0.05

FiO_2_ 1.0-placebo led to a higher RV/LV ratio compared to FiO_2_ 0.21-placebo (p < 0.001), whereas RV/LV ratios in the FiO_2_ 1.0-macitentan group were lower than in the FiO_2_ 1.0-placebo group (p = 0.002). Study groups in FiO_2_ 0.21 did not show significant differences regarding the RV/LV ratio (p = 0.789; Fig. 5).

### Cardiomyocyte cross-sectional area

**Fig. 6:**
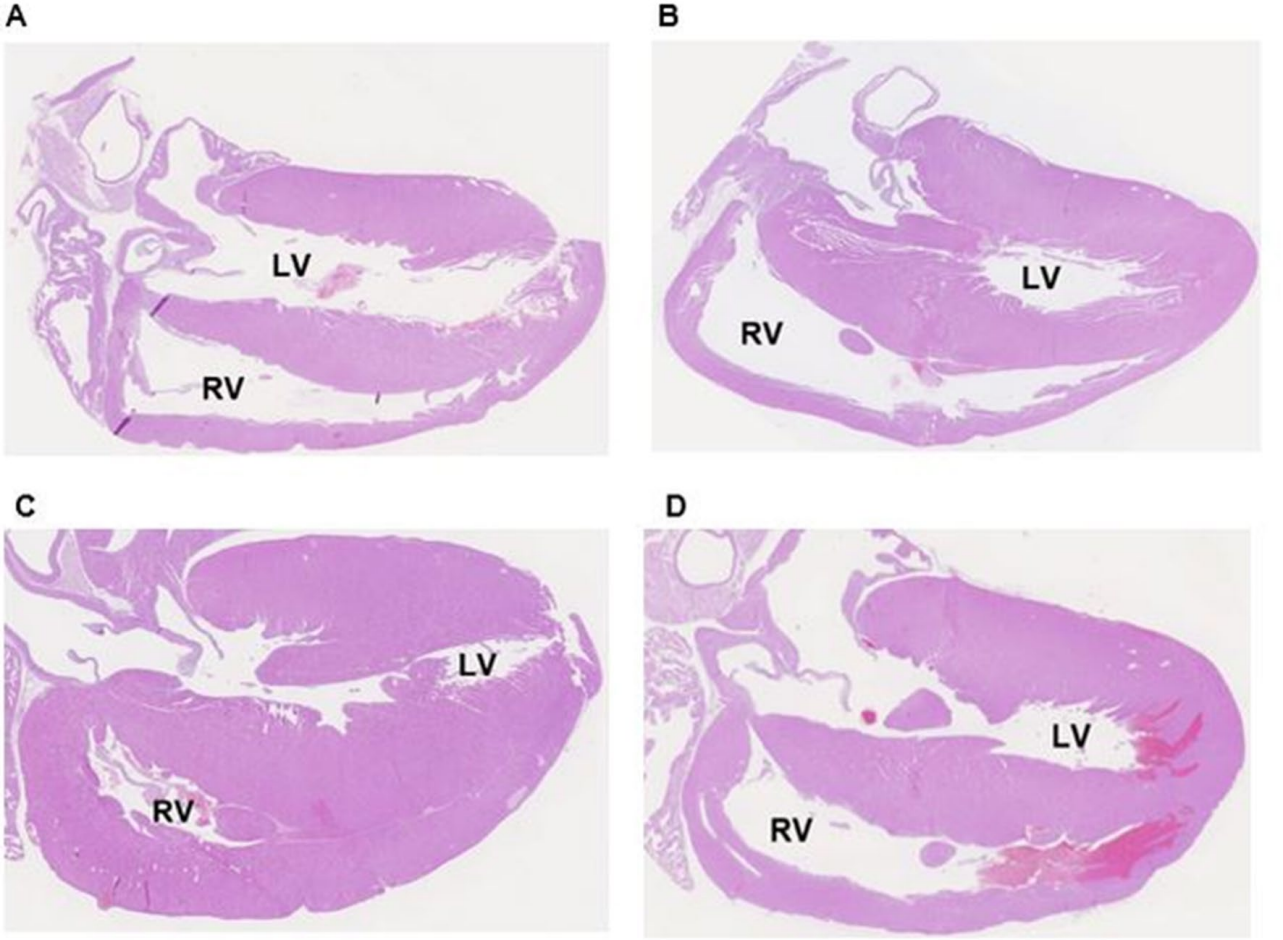
Representative microscopic photographs of heart sections of **A** FiO_2_ 0.21-placebo, (mean RV/LV ratio 0.36; standard deviation 0.02), **B** FiO_2_ 0.21-macitentan (0.35; 0.01), **C** FiO_2_ 1.0-placebo (0.53; 0.03), and **D** FiO_2_ 1.0-macitentan (0.41; 0.03), respectively. RV: right ventricle; LV: left ventricle. Magnification × 1.25

FiO_2_ 1.0 induced larger cardiomyocyte cross-sectional areas compared to FiO_2_ 0.21 in placebo treated animals (p < 0.001). Administration of macitentan in infant rats exposed to FiO_2_ 1.0 did not reduce the cross-sectional area of cardiomyocyte compared to the FiO_2_ 1.0-placebo group (p = 0.651; Fig. 5). Representative H&E microscopic photographs of heart sections: Fig. 6.

### ET-1 plasma concentration

**Fig. 7:**
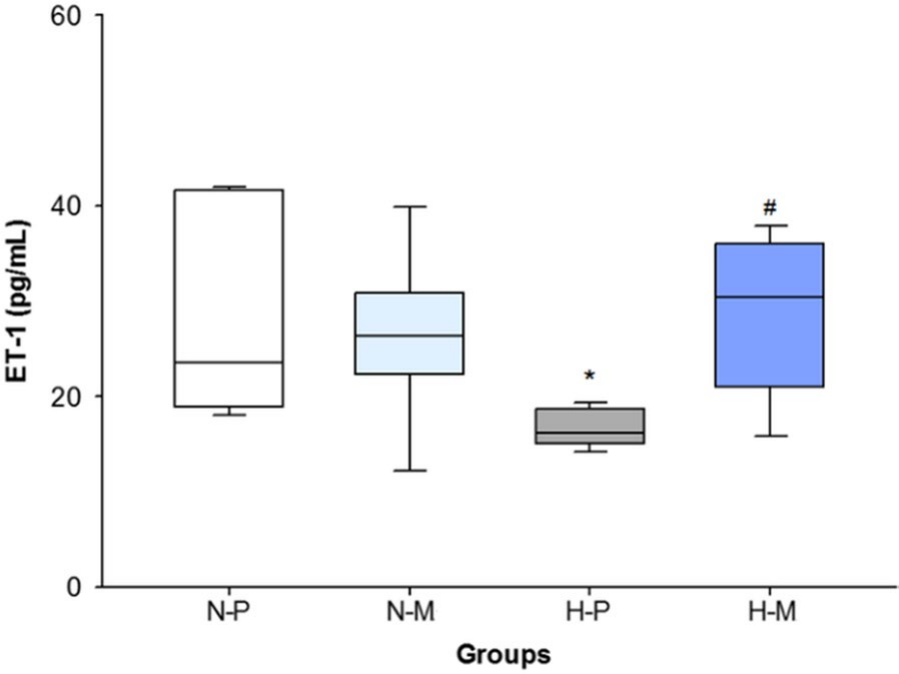
ET-1 plasma concentration in N-P: normoxia (FiO_2_ 0.21)-placebo (n = 6), N-M: normoxia (FiO_2_ 0.21)-macitentan (n = 8); H-P: hyperoxia (FiO_2_ 1.0)-placebo (n = 6); and H-M: hyperoxia (FiO_2_ 1.0)-macitentan (n = 6). Data are expressed as vertical box plots with median, 10th, 25th, 75th, and 90th percentiles. * and ^#^ indicate significant difference to FiO_2_ 0.21-placebo, and FiO_2_ 1.0-placebo, respectively, p < 0.05

The FiO_2_ 1.0-placebo group showed significantly lower ET-1 plasma levels compared with the FiO_2_ 0.21-placebo group (p = 0.019). Administration of macitentan in FiO_2_ 1.0 led to a significantly higher ET-1 concentration compared to FiO_2_ 1.0-placebo (p = 0.015) (Fig. 7).

## Discussion

In this randomized placebo-controlled intervention trial using the hyperoxia rat model for BPD, we were able to demonstrate for the first time that endothelin-1 receptor blockage with the dual ET-1 receptor antagonist macitentan prevented pulmonary vasculature rarefaction and right ventricular hypertrophy. Our hypothesis, that macitentan has the potential to attenuate both alveolar and cardiovascular remodelling was only partly confirmed, as macitentan failed to influence alveolar enlargement and rarefaction, key features of BPD. Since BPD and BPD-PH are results of a number of contributing pathogenicity factors [27], it is unlikely that one single pharmacological intervention changes the whole picture of the disease. However, as treatment options are still sparse, all potential candidates must be carefully evaluated and tested for improvement of outcome.

### Induction of chronic lung disease

We were able to validate our established hyperoxia BPD model [24] through the occurrence of typical histologic features, namely alveolar enlargement and rarefaction, reduction of absolute number of pulmonary vessels, and right ventricular hypertrophy. Further, FiO_2_ 1.0 induced significant elevation of pulmonary arteriole *α*-SMA content, a marker of smooth muscle cell proliferation.

### Effect of ET-1 receptor antagonism on alveolar remodelling

In this study, administration of the ET-1 receptor blocker macitentan did not result in an improvement of structural alveolar remodelling in rat lungs exposed to high fractions of oxygen. This finding was unexpected, because elevated ET-1 protein concentrations, as found in several clinical and translational BPD studies [28–33], seem to contribute to interstitial lung fibrosis via the ET-1 receptor`s central role in pneumocyte collagen deposition [19]. Further, ET-1 stimulates dysregulated angiogenesis, additionally contributing to alveolar simplification [25, 34, 35]. Thus, ET-1 receptor blockage with macitentan should facilitate regular lung development, especially after Gien et al. were able to reverse bleomycin-induced increases in alveolar mean linear intercept by 52% with macitentan`s predecessor bosentan [33].

We speculate that the ongoing pulmonary inflammation driven by long-term high dose oxygen exposure might have outweighed the more specific macitentan effects. It is also possible that the macitentan doses applied in this study were insufficient to counteract all pathophysiologic mechanisms leading to alveolar remodelling.

### Effect of ET-1 receptor antagonism on pulmonary vasculature and right ventricle

Blockage of ET-1 receptors with macitentan in pups exposed to FiO_2_ 1.0 resulted in higher pulmonary arteriole count and in significantly lower *α*-SMA concentrations in the medial wall of pulmonary arterioles in comparison with FiO_2_1.0-placebo. This finding is in agreement with previous experimental studies demonstrating that ET-1 receptor blockage with macitentan promotes pulmonary angiogenesis in rats with pulmonary hypertension [36].

Further, infant rats in the FiO_2_1.0-macitentan group presented significantly lower right to left ventricle diameter ratios compared to FiO_2_1.0-placebo animals. It is likely that macitentan reduced ET-1 receptor-mediated pulmonary vasoconstriction and facilitated regular angiogenesis. Moreover, it is conceivable that macitentan prevented pathologic alterations of pulmonary vascular resistance with increased afterload and right ventricular hypertrophy. This is in line with the study of Iglarz et al. which showed that macitentan treatment of adult rats with bleomycin-induced PH reduced pulmonary artery pressure and prevented right ventricular hypertrophy [37]. Similarly, Valero-Monoz et al. demonstrated that macitentan reversed aldosterone-induced left ventricular hypertrophy in adult C57BL/6J mice [38].

One interesting observation in our study relates to the impact of FiO_2_1.0 on cardiomyocyte cross-sectional area which was not reversed by macitentan although the right ventricular free wall diameter was reduced. There might be two different causal stimuli acting at the same time: First, ET-1, being a potent fibrogenic agent, might have induced proliferation and collagen deposition predominantly in cardiac myofibroblasts [39]. This is especially important when taking into account the change in cardiomyocyte/fibroblast ratio in the developing rat heart during infant and adolescent age where cardiac fibroblasts make up 30% and cardiomyocytes 60% of all heart muscle cells on DOL 1. On DOL 15 this ratio will be reversed with 60% fibroblasts and 30% cardiomyocytes in healthy rats [40]. ET-1 receptor binding could have enhanced this development with an overstimulation of fibroblasts and increased collagen deposition in extracellular matrix. Second, as de Raaf et al. concluded from fetal lamb models and other animal studies, ET-1 receptor binding could be directly involved on cellular level in right ventricular cardiomyocyte hypertrophy, irrespective of pressure overload [41]. The ET-1 system seems to induce a fetal gene programme for right ventricular cardiomyocyte hypertrophy specifically in prenatal cardiac hypertrophy in utero and also in adult cardiac hypertrophy in pulmonary hypertension. In this study this effect on cardiomyocytes might still have been present even under macitentan treatment as exposure to FiO_2_ 1.0 began on DOL 1 whereas treatment with macitentan was initiated on DOL 5.

Therefore, afterload reduction and blockage of RV fibrosis were the two possible reasons for anti-hypertrophic cardiac effects of macitentan. Further, cardiomyocte growth might have been initiated early during the hyperoxic exposure and persisted until DOL 19 without being influenced by macitentan. However, we did not evaluate collagen deposition in fibroblasts or in extracellular matrix.

### Endothelin-1 concentrations

ET-1 concentrations were significantly lower in FiO_2_ 1.0-placebo compared to FiO_2_ 0.21-placebo. This could be a result of hyperoxia-induced enhancement of ET-1 receptor expression [42], resulting in increased peptide binding and a more extensive clearance of ET-1 from plasma. In FiO_2_ 1.0-macitentan, ET-1 concentrations were significantly higher than in FiO_2_1.0-placebo. This finding can be explained by the pharmacological blockage of a sufficient number of ET-1 receptors hampering ET-1 receptor binding and finally leading to an accumulation of ET-1 peptides in the blood stream.

### Macitentan dose

Since the optimal macitentan dosage required for the paediatric population was unknown, we decided to administer 30 mg/kg macitentan by extrapolation from comparable adult rat studies [43] and on the advice of the drug manufacturer (Actelion Pharmaceuticals Ltd, Allschwil, Switzerland). Administration of this medicament by gavage was well tolerated by all infant rats. They showed regular weight gain and no health issues. From our findings with reversal of pulmonary microvasculature rarefaction, reduction of RVH, and increases of ET-1 in plasma, we can conclude, that the dose chosen for this experiment exerted at least some relevant effects. However, absence of side effects with the current dose might allow for even higher macitentan doses potentially enhancing the treatment success.

### Limitations

This study has some limitations. First, we did not perform echocardiographic pulmonary arterial pressure measurement or assessment of right ventricular function to confirm the presence of hyperoxia-induced PH and elevated right ventricular work load. As infant rats are small and echocardiographic evaluations are difficult to perform for reproducible results, we decided to dispense with that. Second, we did not assess ET-1-receptors and receptor-ligand kinetics, which would have been helpful to understand how the concentration of ET-1-receptors and their interaction with ET-1 change in hyperoxia. Third, we did not investigate fibrogenic processes in fibroblasts and myocardial extracellular matrix.

## Conclusions

This was the first study using an in vivo hyperoxia rat model demonstrating the efficacy of the ET-1 receptor antagonist macitentan to counteract hyperoxia-induced BPD-typical pulmonary vascular and right ventricular remodelling with an increase of absolute pulmonary vessel count and reduction of RV/LV ratio. As BPD and its vascular sequelae are very important complications of preterm birth with relevant impact on morbidity, mortality, and health care costs, the results of this study need to be confirmed in further animal studies to pave the way for clinical research in human infants. Furthermore, efficacy and safety of higher macitentan doses have to be examined in future translational dose finding studies.

## Data Availability

The data supporting the conclusions of this article is included within the article.

## Abbreviations

*α*-SMA: *α* Smooth Muscle Actin
BPD: Bronchopulmonary dysplasia
BPD-PH: Bronchopulmonary dysplasia and pulmonary hypertension
BW: Body weight
CO_2_: Carbon dioxide
DAPI: 4′,6-Diamidino-2-phenylindole
DOL: Day of life
ET-1: Endothelin-1
ET_A_: Endothelin-1 receptor subtype A
ET_B_: Endothelin-1 receptor subtype B
FiO_2_: Fraction of inspired oxygen
H&E: Haematoxylin–eosin
IVC: Individual ventilated cages
Lm: Mean linear intercept length
O_2_: Oxygen
ROI: Regions of interest
RVH: Right ventricular hypertrophy
RV/LV ratio: Right ventricular/left ventricular ratio
SD: Sprague Dawley
